# Gamma 3 U-Blade lag screws in patients with trochanteric femur fractures: are rotation control lag screws better than others?

**DOI:** 10.1186/s13018-019-1427-z

**Published:** 2019-12-16

**Authors:** Jehyun Yoo, Sangmin Kim, Junyoung Choi, Jihyo Hwang

**Affiliations:** 1 Department of Orthopaedic Surgery, Hallym University Sacred Heart Hospital, Hallym University School of Medicine, Anyang, Republic of Korea; 2 Department of Orthopaedic Surgery, Korea University Guro Hospital, Seoul, Republic of Korea; 3 Department of Orthopaedic Surgery, Gangnam Sacred Heart Hospital, Hallym University College of Medicine, 1 Singil-ro, Yeongdeungpo-gu, Seoul 07441 Republic of Korea

**Keywords:** ITST, PFNA II, Gamma 3, Trochanteric fracture

## Abstract

**Background:**

Intramedullary hip nails may be classified as blades or screws depending on the type of lag screw used. Recently, a combination of lag screw types with a U-clip insertion has also been used. The purpose of this study was to evaluate the clinical and radiological outcomes of these new screw types.

**Methods:**

A total of 185 patients with trochanteric femoral fractures (age ≥ 65 years) who underwent surgery with intramedullary nails were selected. Surgeries with InterTrochanteric/SubTrochanteric (ITST), Proximal Femoral Nail Antirotation (PFNA), and Gamma 3 U-Blade lag screws were performed between January 2011 and June 2016. The AO/OTA classification, presence of a basicervical fracture type on 3D-CT, BMI, BMD, reduction quality, position of the lag screw, TAD (tip apex distance) of the lag screw, sliding distance of the lag screw, varus change (neck shaft angle), radiological union period, fixation failure and functional outcome as determined by walking ability were analyzed.

**Results:**

There were 3/60 (5.0%) cases of fixation failure in the ITST group, all caused by cut-out; 4/57 (7.0%) in the PFNA II group: 3 caused by cut-through and 1 by metal fracture; 1/68 (1.5%) in the Gamma 3 U-Blade lag screw group (*P* = 0.301). In each group, the sliding distance of the lag screw showed a significant difference (*P* = 0.017), whereas significant sliding over 10 mm showed no statistically significant results.

**Conclusion:**

There was only one (1.5%) case of fixation failure in the Gamma 3 U-Blade lag screw group. The sliding distance of the U-Blade was found to be in the middle, between the PFNA II (shorter) and ITST (longer) implants. The new rotational control lag screw seems to be comparable to other screw types.

## Background

Hip fractures are the third most common type of fractures after distal radius fractures and hand fractures. Hip fractures are the most common type of fracture among elderly people. In particular, the prevalence of trochanteric fractures compared with femoral neck fractures has gradually increased. The average age of patients with these types of fractures is also gradually increasing. Unlike other fractures, trochanteric fractures can be fatal and, thus, they deserve special care and attention from orthopedic surgeons [1]. Using intramedullary nails during osteosynthesis has also been reported to be biomechanically superior to the use of compression hip screws [2]. InterTrochanteric/SubTrochanteric (ITST, Zimmer Biomet, Warsaw, IN, US) and Proximal Femoral Nail Antirotation (PFNA, Synthes, Paoli, Switzerland) screws are commonly used options for intramedullary nailing, and they might be representative of the blade type and screw type of lag screws. The Gamma nail has been improved and used in Asian patients who have smaller body sizes (170 mm) as a fourth-generation Gamma nail (Asia–Pacific and Japanese versions). After that, the Gamma 3 nail model designed with the use of a U-Blade lag screw was developed to provide improved outcomes when treating patients with unstable proximal femur fractures, and for those at high risk of rotation, this model is the newest model in the Gamma series. So far, there have been limited results for this newest type of Gamma nail [3, 4].

**Fig. 1:**
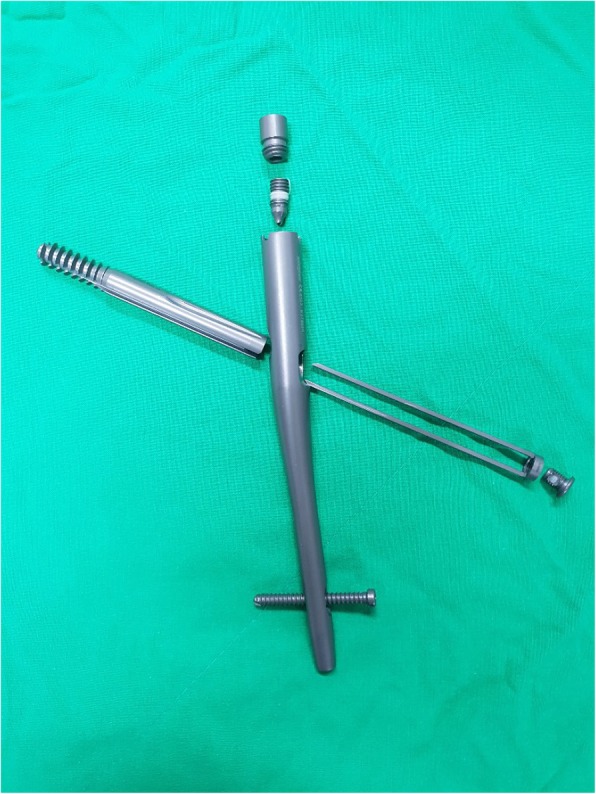
Photograph of rotation control (RC) of the Gamma 3 screw (Stryker, Schonkirchen, Germany)

**Fig. 2:**
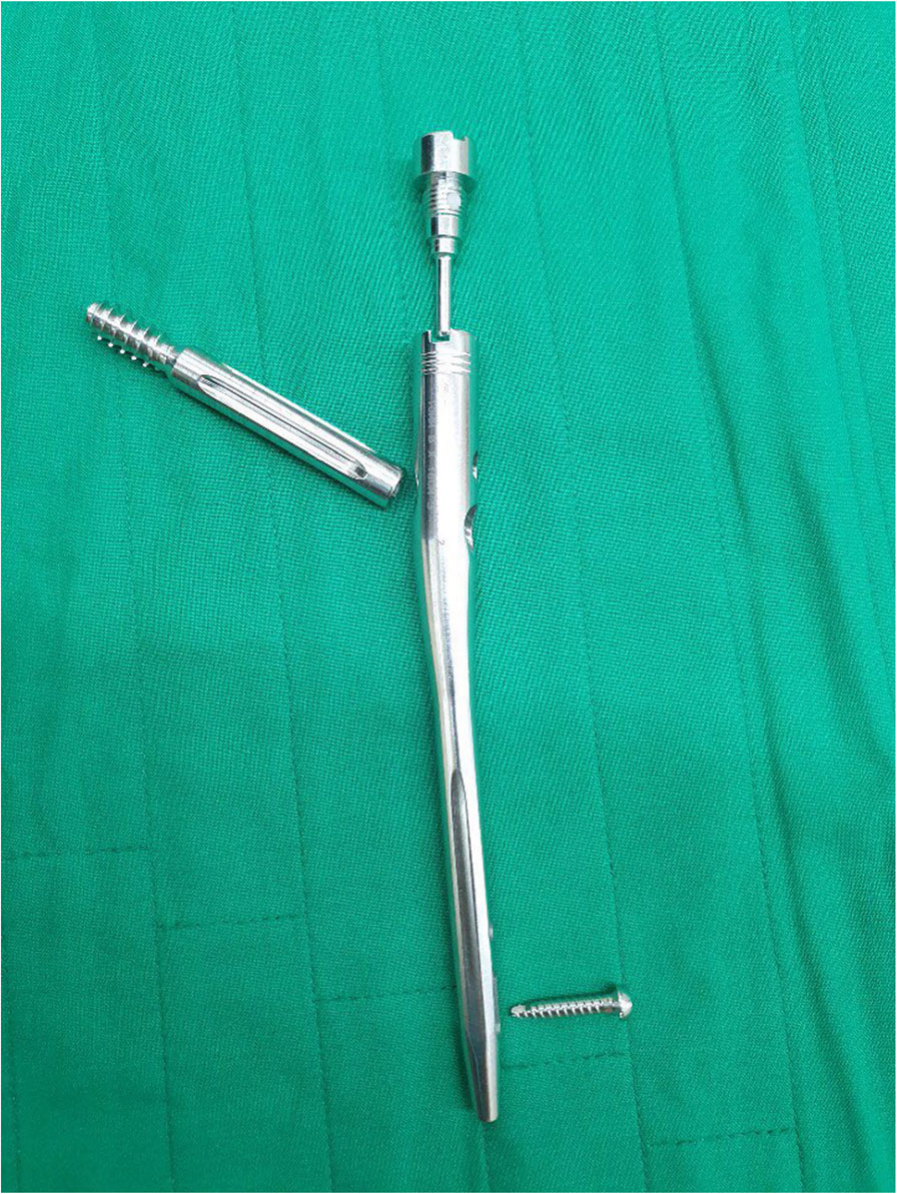
Photograph of PFNA (Proximal Femoral Nail Antirotation, Synthes, Paoli, Switzerland)

**Fig. 3:**
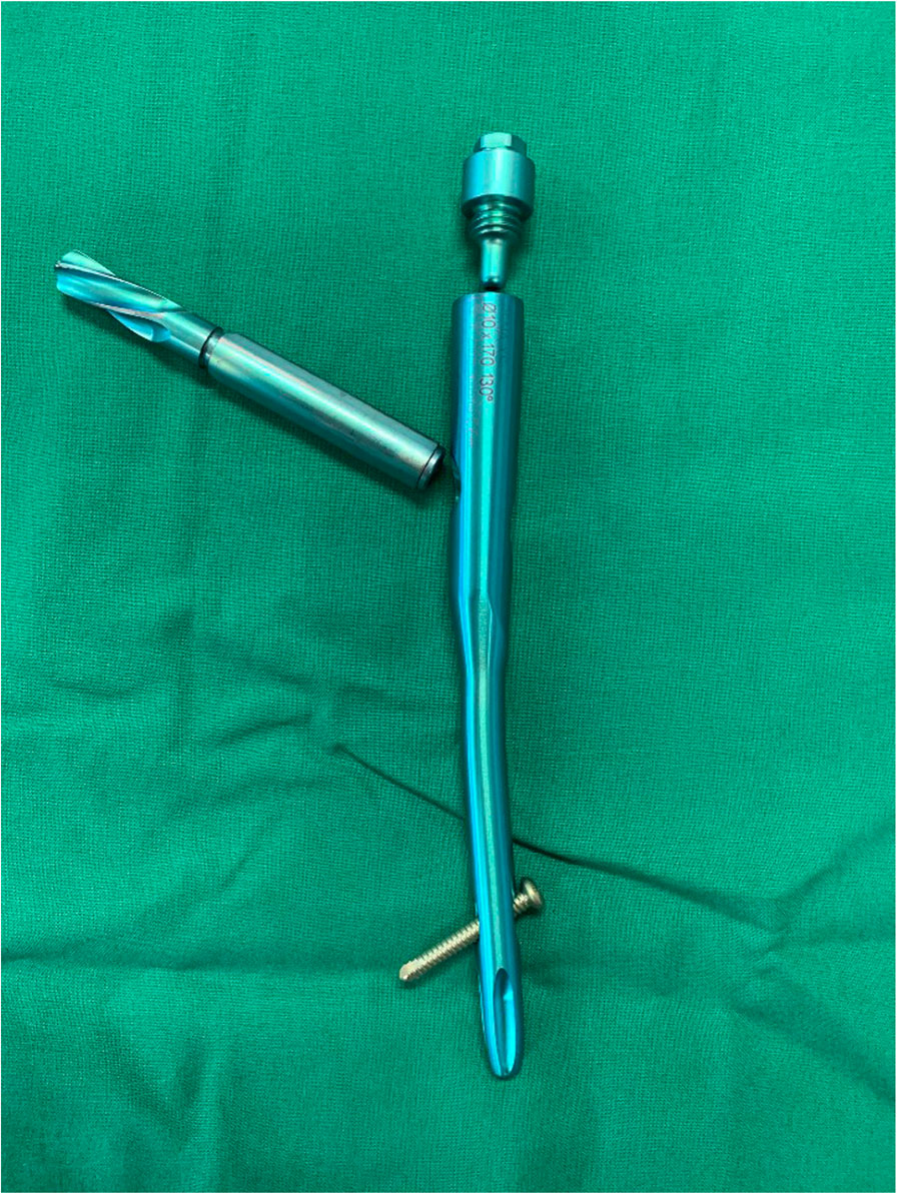
Photograph of InterTrochanteric/SubTrochanteric (ITST), Zimmer Biomet, Warsaw, IN, US)

In this study, we sought to compare radiological and functional outcomes, including implant-related complications, among three different types of screws: the most recently developed rotation control (RC) Gamma 3 screw (Stryker Trauma GmbH, Schoenkirchen, Germany) (Fig. 1), the latest PFNA II blade lag screw (Fig. 2) and a traditional InterTrochanteric/SubTrochanteric (ITST) lag screw (Fig. 3).

## Methods

### Study design

This study was approved by the local Institutional Review Board. We obtained consent from all patients with permission to operate. All the data were analyzed by retrospectively reviewing the medical charts, X-rays and phone interviews in terms of the functional outcomes.

### Inclusion and exclusion criteria

We included patients with at least 12 months of follow-up between January 2011 and June 2016 who could walk on their own. Patients aged 65 years or older were selected. Basicervical types of trochanteric fractures were included. The exclusion criteria were as follows: patients who could not walk on their own prior to the injury, patients who had other pathological fractures due to causes other than osteoporosis, patients who died during the follow-up, patients with high-energy injuries such as motor vehicle accidents or falling from a height, and patients who had less than 12 months of follow-up. The average follow-up periods for patients with ITST, PFNA II, and Gamma 3 RC nails were 28.5 (12–53), 37 (12–143), and 14.8 (12–28) months, respectively. The types of implants were randomly chosen, and the use of the Gamma 3 U-Blade lag screw started in 2014, as it was introduced in this period.

### Fracture classification

Fractures were classified by two physicians according to the AO/OTA classification [5]. All fractures were also analyzed using preoperative CT imaging to determine the configuration of fracture patterns, the presence of greater trochanter comminution and whether the fracture was of a basicervical fracture type. This fracture type was defined as a partial capsular fracture, which can be a variant of a trochanteric fracture. This fracture type corresponds to the A1 fracture type on the AO/OTA classification.

### Analyzed variables

Altogether, there were 60 cases of ITST nails, 68 of Gamma 3 RC nails, and 57 of PFNA II nails. We also compared the body mass index (BMI), bone mineral density (BMD), degree of fracture reduction, position of the lag screw in the femoral head, tip apex distance (TAD), sliding distance of the lag screw, changes in the neck shaft angle, radiological bone union period, and fixation failure and its causes. For the functional outcomes, the ability status was evaluated by Koval grade [6, 7] (Table 1). Recovery rates were calculated as the proportion of the patients who obtained the preinjury ambulatory status.

**Table 1:** Categories of ambulatory ability

1. Independent community ambulator	
2. Community ambulatory with cane	
3. Community ambulatory with walker/crutches	
4. Independent household ambulator	
5. Household ambulatory with cane	
6. Household ambulatory with walker or crutches	
7. Nonfunctional ambulator^a^	

^a^Used only for after-fracture ambulation

### Surgical procedure

The subjects were asked to assume a supine position on the fracture bed and received general or spinal anesthesia. After fixation in the fracture bed, manual reduction was performed through traction and internal rotation and/or adduction. Satisfactory results were confirmed via fluoroscopy. When the manual reduction was unsatisfactory, a long Kelly, Hohmann retractor or bone hook was used to compress the lateral or anterior cortex and to pull the medial cortex for reduction. No invasive open reduction was performed. We attempted manual reduction to maintain the continuity of the medial and anterior cortices. After manual reduction, an entry point was designated, a guide pin was inserted, and proximal reaming was performed in the proximal area using a conical reamer that was thicker than the nail. When a fracture gap remained, we reduced it using a compression technique for each implant. The distal fixation screw was fixed in a static locking mode regardless of the fracture type in all the patients based on the surgeon’s preference. Approximately 2–3 days postoperatively, when the patients could tolerate weight-bearing in a sitting position, the patients were asked to try to stand using a tilt table. Walking was allowed when the pain became tolerable. Restricted weight-bearing was taught and initiated by touching approximately 20 kg on a scale; the patients were allowed to walk using the parallel bar or rolling walker. Various weight-bearing training exercises were performed not based on the reduction or bone quality but only based on the subject’s pain level and medical condition. All rehabilitation programs were conducted equally in all patients. All the patients were discharged or transferred with proper walking aids, such as wheelchairs or walkers.

### Outcome parameters

Cut-out was defined as penetration through the femoral head that was visible on X-ray, while cut-through was defined as the perforation of the femoral head from centric movement and without lateral movement by the lag screw. The lag screw position in the femoral head was measured from the anteroposterior (AP) and lateral images. The general rule was to measure the position from the final follow-up X-ray images. Centric position was defined as a screw positioned in the center of both the AP and lateral images, while eccentric position was defined as the screw deviating from the center on any of the views. Reduction quality was also evaluated based on radiological evidence. Maintaining continuity of the medial cortex on AP view, the anterior cortex in the lateral view was defined as anatomical reduction. Successful reduction in both views was defined as anatomical reduction, whereas continuity not maintained in any of the views was defined as non-anatomical reduction. The sliding distance and neck shaft angle, which can represent the shortening of and varus change in the fracture site, were calculated as differences between the initial X-ray and final X-ray. All the parameters obtained in the X-ray were adjusted by the calibration of the magnification of the real size and angle of the inserted implants. Significant sliding of the lag screw was defined arbitrarily by the authors, as an irritation sign due to the prominent lateral impingement without deep or superficial infection, as a lag screw sliding distance ≥ 10 mm in the plain X-ray or the removal of the lag screw due to one or both reasons. The excessive angular change was also arbitrarily defined as 10° or more. For the union, a clinical union was determined when the pain scale improved during the follow-up period and with the absence of pain or tenderness during weight-bearing. Radiological union was defined when the fracture line was lost and when three or more external or internal calluses were formed in the anteroposterior and lateral radiological images taken during the follow-up period [8]. For the analysis of fracture types and radiologic union, three orthopedic surgeons performed the measurements with an interval to minimize interobserver error.

### Complications

For implant-related complications, screw cut-out, cut-through and back-out, as well as nail or screw breakage, were analyzed. Other medical complications (e.g., infection, pressure ulcers, pneumonia, urinary tract infections, delirium, cardiovascular complications, and deep vein thrombosis) were also investigated but not statistically analyzed.

### Statistical analysis

All continuous variables were analyzed for normality by the one-sample Kolmogorov–Smirnov test. For the continuous variables with normal distribution, such as BMI and the varus change in the neck shaft angle, *t* test was performed. For continuous variables without a normal distribution, such as age, sliding distance and TAD, the Kruskal–Wallis test was performed. The other categorical variables, such as sex, classification, reduction quality, and complications, were analyzed by Pearson’s Chi-square test. Statistical analysis was performed using IBM SPSS (Version 25, Chicago, IL). Statistical significance was defined as a *P* value less than 0.05.

## Results

### ITST (*n* = 60)

**Fig. 4:**
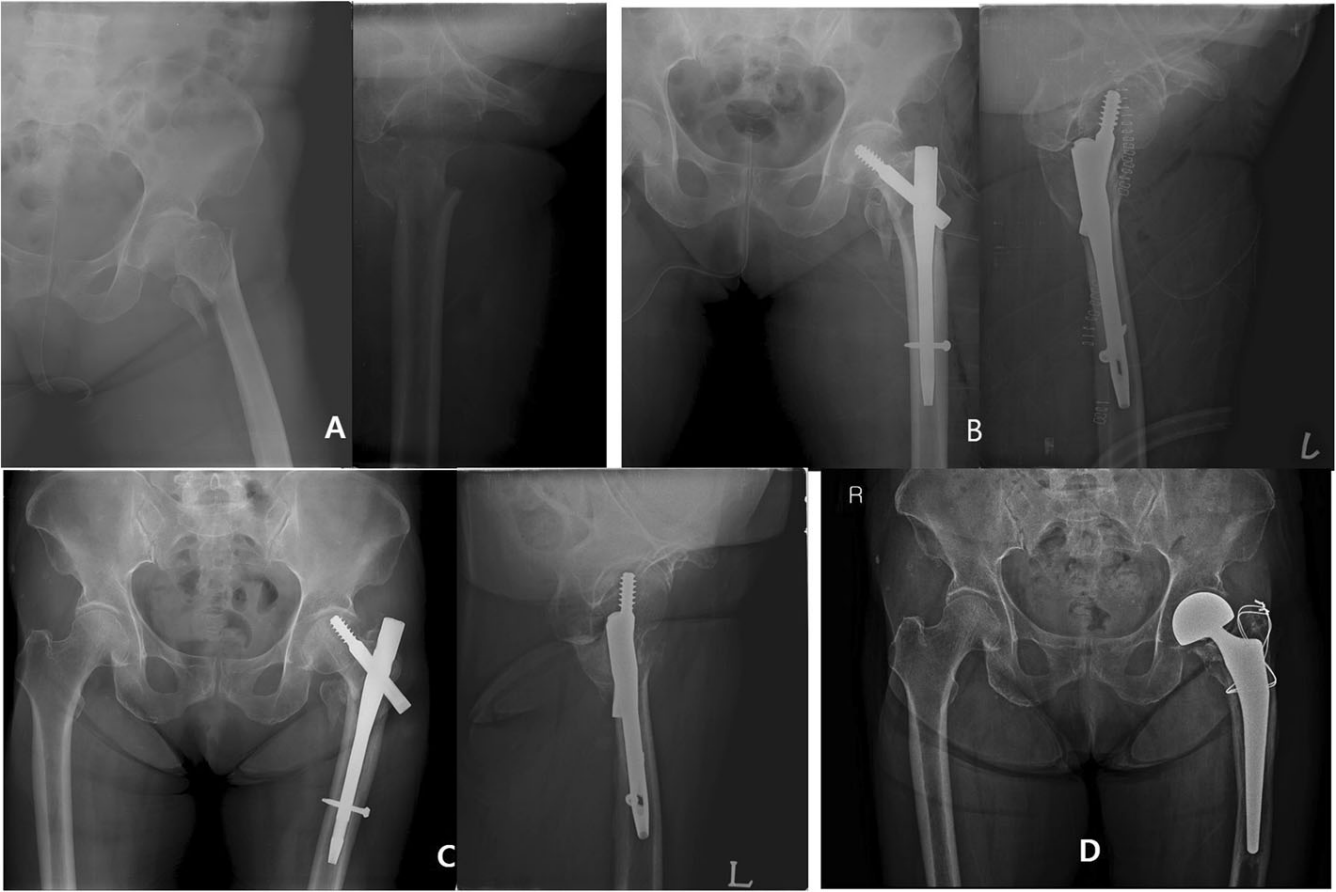
A 70-year-old woman with an A22 fracture and a large posteromedial fragment on the day of the trauma (**a**). She underwent ITST surgery (**b**). The lag screw demonstrated cut-out 3 months postoperatively (**c**). Bipolar hemiarthroplasty was performed (**d**)

The mean age was 78.5 ± 7.0 years, with a female-to-male ratio of 44 to 16. The BMI was 22.8 ± 3.9, and the *T* score of BMD was − 2.6 ± 1.3. A total of 38 cases were classified as 31A1 based on AO/OTA on the preoperative X-ray, 6 were basicervical fracture types on 3-D CT imaging, and 37 were comminuted in the greater trochanter. Fifty-seven (95%) patients gained union. Union was gained at an average of 18.7 weeks (12–40 weeks). The TAD was 19.9 ± 0.98 mm. The sliding distance was 5.6 ± 3.6 mm, and three cases migrated over 10 mm. The change in the neck shaft angle was 2.3° ± 6.08; four cases were over 10°. The position of the lag screw was centric in 45 cases, and 43 cases were anatomically reduced. Three cases demonstrated a cut-out lag screw. One case is shown in Fig. 4. The mean Koval grade at the final visit was 2.9 (1–7). The overall recovery rate of ambulatory status was 46.5%.

### PFNA II (*n* = 57)

**Fig. 5:**
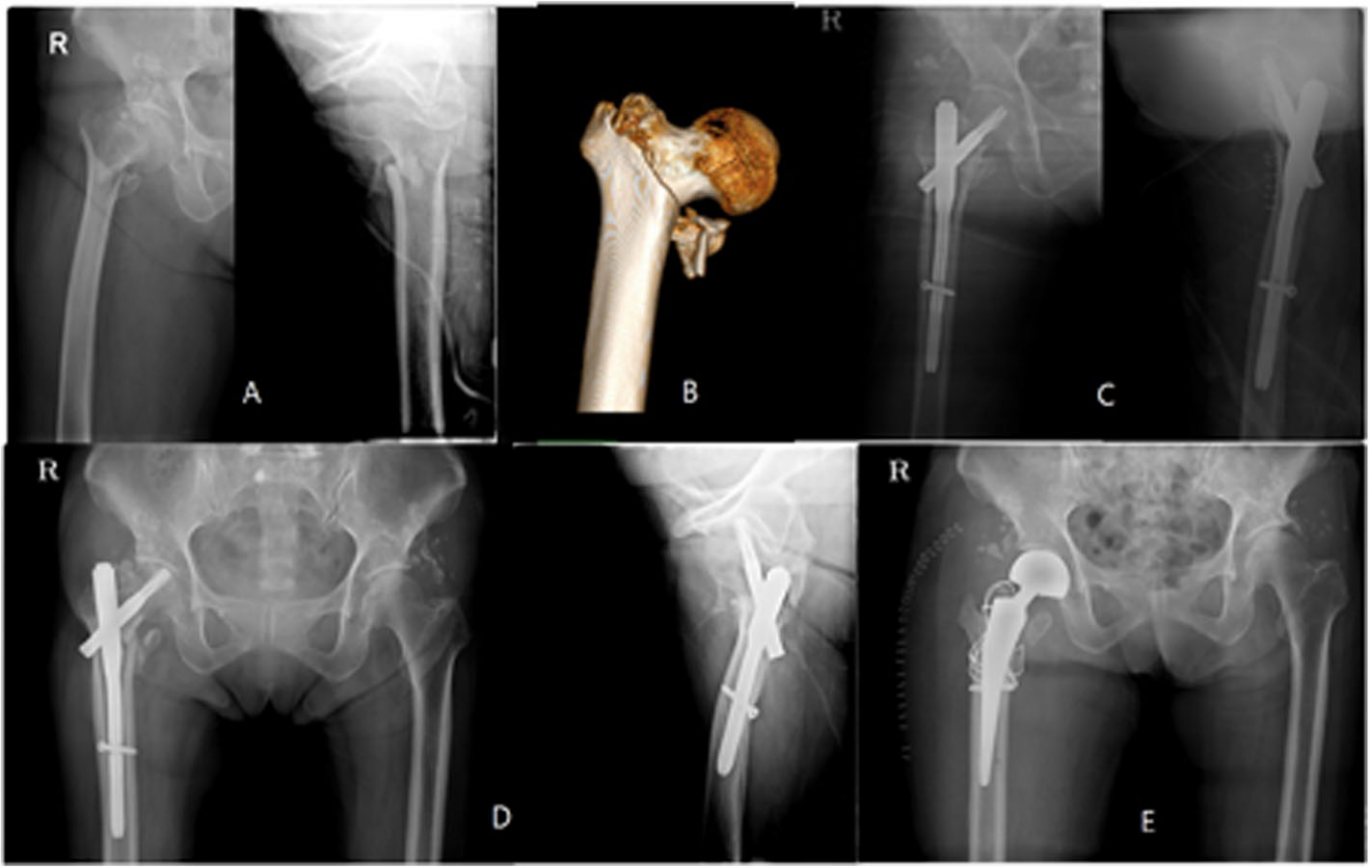
An 81-year-old woman fell on the ground; the proximal fragment was short with varus angulation (**a**). 3D-CT imaging showed a basilar neck fracture (**b**). The fragment was reduced using PFNA II screws (**c**). The lag screw demonstrated cut-through 3 months postoperatively (**d**). We converted it via hemiarthroplasty (**e**)

The mean age was 79.5 ± 7.0 years, with a female-to-male ratio of 42 to 16. The BMI was 21.4 ± 1.4, and the *T* score of BMD was − 2.8 ± 1.4. A total of 24 cases were type 31A1, and 31 cases were classified as A2 on AO/OTA on the preoperative X-ray. Five cases were basicervical fracture types on 3-D CT imaging, and 37 cases were comminuted in the greater trochanter. Fifty-three patients (92.8%) out of 57 gained complete union. The average union was gained at 17.6 weeks (12–24 weeks). The TAD was 19.2 ± 5.02 mm. The sliding distance was 3.3 ± 3.6 mm, and there was no excessive migration. The change in the neck shaft angle was 1.3° ± 1.20. Two patients showed excessive varus change over 10°. The position of the lag screw was centric in 43 cases, and 40 cases were anatomically reduced. Four cases had nail complications: three were cut-through, and one was nail breakage. The cut-through case is shown in Fig. 5. The mean Koval grade at the final visit was 3.1. The overall recovery rate of ambulatory status compared to the preoperative status was 46.8%.

### Gamma 3 U-Blade (n = 68)

**Fig. 6:**
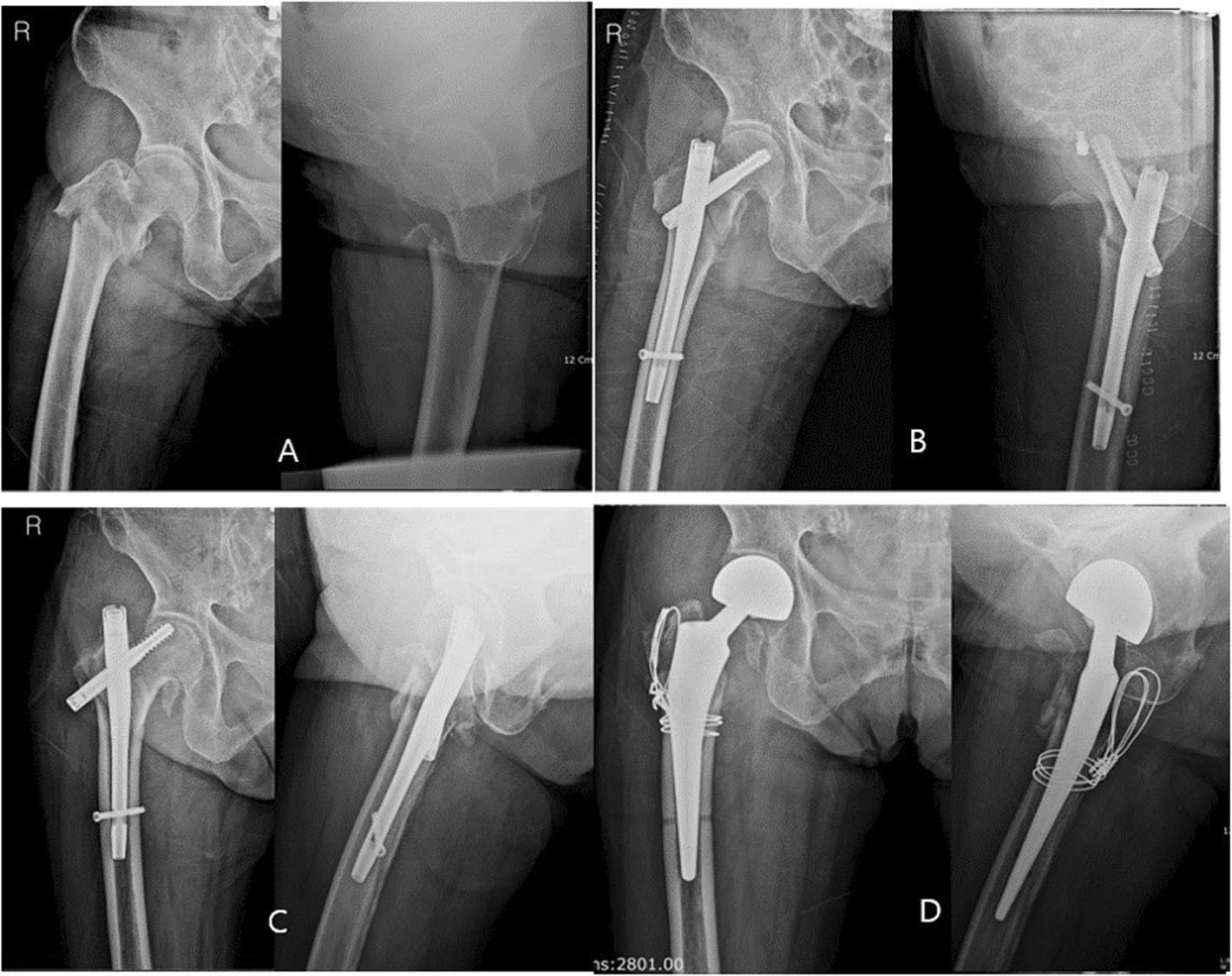
An 83-year-old woman suffered from a fall from a height at home; the fracture type was reverse oblique (A32) (**a**). The fragment was reduced using a Gamma 3 RC screw (**b**). The lag screw demonstrated cut-out of the head 3 months postoperatively (**c**). We converted it via hemiarthroplasty (**d**)

The mean age was 79.2 ± 7.5 years, with a female-to-male ratio of 51 to 17. The BMI was 22.2 ± 3.9, and the *T* score of BMD was − 2.7 ± 1.2. A total of 21 cases were 31A1 type, 41 cases were A2, and 6 cases were A3 on AO/OTA on the preoperative X-ray. A total of 9 cases were basicervical fracture types on 3-D CT imaging, and another 39 cases were comminuted in the greater trochanter. One case of delayed union and one of nonunion due to screw cut-out are shown. One patient with delayed union obtained complete union without the need for any additional procedure at 36 weeks after the operation. The union rate was 98.5%, with an average of 19.9 weeks (12–36 weeks). The TAD was 18.1 ± 4.45 mm. The sliding distance was 3.8 ± 3.1 mm, and there were three cases of excessive migration. The change in the neck shaft angle was 2.2° ± 5.46. A total of 3/68 cases showed a change of 10° or more. The position of the lag screw was centric in 53 cases, and 53 cases were anatomically reduced. One case is shown as a cut-out of the femoral head (Fig. 6). The mean Koval grade at the final visit was 2.7 (1–7). The overall recovery rate of ambulatory status was 49.8%. The statistically compared data are presented in Table 2.

**Table 2:** Comparative statistical analysis of three groups

	ITST (*n* = 60)	PFNA (*N* = 57)	U-Blade (*n* = 68)	*P* value
Age (years)	78.5 ± 7.0	79.5 ± 7.0	79.2 ± 7.5	0.187
Gender (female:male)	44:16	42:15	51:17	0.977
AO classification				
31 A1	38	24	21	1.000
31 A2	22	31	41	
31 A3	0	2	6	
BMI (kg/m^2^)	22.8 ± 3.9	21.4 ± 3.9	22.2 ± 3.9	0.162
BMD (*T* score)	− 2.6 ± 1.3	− 2.8 ± 1.4	− 2.7 ± 1.2	1.000
Basicervical fracture type on 3D-CT	6 (10%)	5 (8.7%)	9 (13.2%)	0.145
GT comminution on 3D-CT	37 (61.7%)	37 (64.9%)	39 (57.4%)	0.684
TAD of lag screw (mm)	19.9 ± 0.98	19.2 ± 5.02	18.1 ± 4.45	0.835
Sliding distance of lag screw (mm)	5.6 ± 3.6	3.3 ± 3.6	3.8 ± 3.1	0.017
Excessive sliding over 10 mm	3	0	3	0.247
Varus change (°)	2.3° ± 6.08	1.3° ± 1.20	2.2° ± 5.46	0.762
Excessive change over 10°	4	2	3	0.634
Position of lag screw				
Centric	45	43	53	0.914
Eccentric	15	14	15	
Reduction quality				
Anatomical	43	43	53	0.572
Non-anatomical	17	14	15	
Fixation failure	3 (5.0%)	4 (7.0%)	1 (1.5%)	0.301
Cause of failure				
Cut-out	3	0	1	0.092
Cut-through	0	3	0	
Nail breakage	0	1	0	
Walking ability recovery (Koval grade)	52.7%	46.8%	49.8%	0.732

## Discussion

Rotation of the proximal fragment or lag screw is related to the initiation of screw migration and consequent fixation failure [9], so many efforts for lag screw design, especially the prevention of rotation, have evolved. Proximal Femoral Nail Antirotation (PFNA) and Gamma 3 U-Blade screws represent some of the most evolved models for the control of the early motion of lag screws, such as toggling, rotation, and migration. Although our study did not reveal differences in clinical results, lag screw migration in terms of the sliding distance showed a significant difference, and these rotation control lag screws can be superiorly resistant to lag screw migration. Although only a slight difference was shown in our study, it suggests that the rotation control lag screw might be more effective than other types of screws.

We were unable to find any statistically significant differences among the three different types of nail systems. ITST nails had a greater sliding distance than the other two nailing systems. However, sliding distance alone does not affect surgical outcomes or prognosis. It should be noted that metal irritation due to sliding distance or metal removal may result in an excessive sliding distance. Sliding distance can also represent the migration of lag screws, and it can be a potential risk factor for lag screw-related complications. The newly designed Gamma 3 RC lag screw is thought to have few implant-related complications. We found that cut-through was the most common complication among the blade types, while cut-out was most common among the screw types. As is the case with numerous previous studies, it is difficult to predict treatment outcomes based on screw design, but the latest implants may prevent further complications. In terms of the factors of fixation failure for surgeons, the implant choice can affect the clinical outcomes when the fracture configurations look unstable. Gamma 3 U-Blade screws can be a good option for trochanteric hip fractures in the clinical field.

The CT scan is a popular and beneficial tool for the preop or postop evaluation of hip fractures. In the current study, a CT scan was routinely performed for more information about the fracture configurations. CT scans are also good tools for the evaluation of coronal fracture patterns [10]. A new classification for CT scans has been proposed, indicating the significance of CT scans for hip fractures [11, 12]. The extension of fracture lines to the greater trochanter area and fracture levels, such as with basicervical fracture types, might affect the reduction quality of intramedullary nailing, so we also analyzed these factors. The comminution of the greater trochanter did not have any significance, but the fracture level for basicervical type fractures was related to fixation failure in this study. A basicervical type fracture can be considered an unstable fracture in intramedullary nailing. In other words, it can be thought of as stable from the perspective of femoral neck fractures but unstable from the perspective of trochanteric fractures. A good example would be a fracture with a pistol grip deformity that shows significant varus angulation. In our study, 30.3% (56/185) of cases showed this type of fracture on CT scans. Among patients in the fixation failure groups, 62.5% (5/8) showed this type of fracture.

There are still controversies surrounding studies comparing intramedullary nail systems. There is a wide variety of studies comparing blades versus screws and the use of one screw versus two screws [13–20]. Researchers have been unable to find any biomechanical or clinical differences between the different screw designs (screw type versus blade type). Unlike previous studies, we included Gamma 3 RC lag screws, the latest model among one-screw types, and PFNA II, the latest model among blade types, as well as ITST, which was the popular conventional screw design, to determine the advantages and disadvantages among these three different types of implants. Since the latest Gamma 3 RC lag screw type model has not been compared often, including it in the comparison was even more meaningful. According to Lang et al. [3], when conventional nails and the newly designed RC screw were compared in cases of unstable trochanter fractures, cut-out complications did not decrease with either design. We could not show that Gamma 3 RC lag screws were superior. Only a few studies have compared three or more nailing systems. Ma et al. [21] reported that InterTan, a two-screw type, was superior to PFNA II and Gamma 3 nails, which are one-screw types. Several papers comparing two-nail systems have already been published [22, 23], and the clinical outcomes from each of these nails have been reported [23–25]. However, to our knowledge, this is the first study to compare three one-screw types.

We acknowledge that there are major limitations in this study. First, there were a limited number of patients and a difficult follow-up because the patients were geriatric. If patients died or could not be followed properly, treatment outcomes could not be fully evaluated. Therefore, the results might not be sufficiently suitable for statistical analysis. Second, because the Gamma 3 RC lag screw is the latest model, this group had a shorter follow-up period than the other groups. However, implant-related complications are usually determined within 3 months; thus, shorter overall periods of follow-up might not be affected in the current study. Last, the data from X-rays, especially the neck-shaft angle and sliding distance, can deviate due to the observers or X-ray evaluations.

## Conclusions

PFNA II, Gamma 3 U-Blade and ITST nails can be a good option for the treatment of trochanteric fractures. The sliding distance, which can be an indicator of lag screw migration and cut-out, was superior in the groups with rotation control lag screws (PFNA II and Gamma 3 U-Blade) than in the ITST group. Altogether, the results show that the new rotation control lag screw is not superior to the other types of screws. The numerical complication rate was lower, and the sliding distance, which might be related to implant-related complications, was shorter for the rotation control lag screws than for the conventional lag screws (ITST). Additional studies are necessary to determine whether better clinical results are achieved with lower fixation failure rates or whether improved function is achieved with rotation control lag screws such as Gamma 3 for trochanteric femur fractures.

## Abbreviations

ITST: InterTrochanteric/SubTrochanteric
PFNA: Proximal Femoral Nail Antirotation
RC: rotation control
